# Maternal and perinatal adverse outcomes in women with pre-eclampsia cared for at facility-level in South Africa: a prospective cohort study

**DOI:** 10.7189/jogh.08-020401

**Published:** 2018-12

**Authors:** Hannah L Nathan, Paul T Seed, Natasha L Hezelgrave, Annemarie De Greeff, Elodie Lawley, Frances Conti-Ramsden, John Anthony, Wilhelm Steyn, David R Hall, Lucy C Chappell, Andrew H Shennan

**Affiliations:** 1 Department of Women and Children’s Health, King’s College London, London, UK; 2 Department of Obstetrics and Gynaecology, University of Cape Town, Cape Town, South Africa; 3 Department of Obstetrics and Gynaecology, Stellenbosch University, Cape Town, South Africa

## Abstract

**Background:**

Hypertensive disorders of pregnancy contribute to 14% of all maternal deaths, the majority of which occur in low- and middle-income countries. The aim of the study was to describe the maternal and perinatal clinical outcomes of women with pre-eclampsia living in middle- and low-income countries.

**Methods:**

The study was a prospective observational study of women with pre-eclampsia (n = 1547, 42 twin pregnancies) at three South African tertiary facilities. Using stepwise logistic regression model area under the receiver operating characteristic curve (AUROC) values, the association between maternal baseline and admission characteristics and risk of adverse outcomes was evaluated. Main outcome measures were eclampsia, kidney injury and perinatal death.

**Results:**

In 1547 women with pre-eclampsia, 16 (1%) died, 147 (9.5%) had eclampsia, four (0.3%) had a stroke and 272 (17.6%) had kidney injury. Of the 1589 births, there were 332 (21.0%) perinatal deaths; of these, 281 (84.5%) were stillbirths. Of 1308 live births, 913 (70.0%) delivered <37 completed weeks and 544 (41.7%) delivered <34 weeks’ gestation. Young maternal age (AUROC = 0.76, 95% confidence interval (CI) = 0.71-0.80) and low Body Mass Index BMI (AUROC 0.65, 95% CI = 0.59-0.69) were significant predictors of eclampsia. Highest systolic blood pressure had the strongest association with kidney injury, (AUROC = 0.64, 95% CI = 0.60-0.68). Early gestation at admission was most strongly associated with perinatal death (AUROC = 0.81, 95% CI = 0.77-0.84).

**Conclusions:**

The incidence of pre-eclampsia complications, perinatal death and preterm delivery in women referred to tertiary care in South Africa was much higher than reported in other low- and middle-income studies and despite access to tertiary care interventions. Teenage mothers and those with low BMI were at highest risk of eclampsia. This information could be used to inform guidelines, the research agenda and policy.

Approximately 800 women die every day from problems related to pregnancy and childbirth [1], with hypertensive disorders of pregnancy causing 14% of all maternal deaths and pre-eclampsia contributing to 500 000 perinatal deaths annually [2,3]. In South Africa, with provision of high-level tertiary care, hypertensive disorders of pregnancy are the second leading cause of maternal death [4,5].

Over the last century, maternal mortality rates in high-income countries have steadily declined. Now, almost all maternal deaths occur in low- and middle-income countries (LMICs) [6]. In 2015, the lifetime risk of maternal death in high-income countries was 1 death per 6000 pregnant women compared to 1 death in 36 in sub-Saharan Africa [7]. In high-income countries, maternal mortality from pre-eclampsia declined most dramatically between 1940 and 1970, with a 90% reduction in eclampsia. This is largely due to improved antenatal care and better access to timely delivery [8]. In LMIC settings women continue to die from preventable complications of pre-eclampsia. It is unclear whether routine pregnancy care or emergency obstetric care should be primarily targeted as strategies for improving outcomes.

This study aimed to describe the maternal and perinatal clinical outcomes of pre-eclamptic women treated in tertiary facilities in South Africa, a middle-income country setting where appropriate medical care is available. The study also aimed to evaluate the association between maternal baseline and admission characteristics and the risk of four severe clinical outcomes.

## METHODS

This prospective observational cohort study was undertaken between January 2015 and May 2016 at three state tertiary level maternity units in South Africa (Groote Schuur, Tygerberg and Kimberley Hospitals). Women with a clinical diagnosis of pre-eclampsia determined by the woman’s health care provider during their admission (either antepartum or postpartum) were included in the study. There were no exclusion criteria.

Clinicians managed women according to local practices. Blood pressure (BP) measurements were taken using the Microlife CRADLE Vital Signs Alert (VSA), a BP device validated for use in pregnancy, including pre-eclampsia [9]. Almost all existing BP devices were replaced with the VSA in the three maternity units; BP monitoring systems within anaesthetic and recovery machines could not be replaced.

Data were extracted through patient notes reviewed by a local researcher at each site. Data quality checks were undertaken on the database by a subsequent researcher and any disagreement was resolved by a medically trained adjudicator. Women with missing data were included but not analysed for the variable for which the data were missing.

Frequencies were used to describe maternal baseline and admission characteristics and to describe the proportion of women affected by maternal, perinatal and process measure adverse clinical outcomes. At the time of designing the study, a core outcome set for pre-eclampsia or hypertension in pregnancy did not exist. The principal clinical outcomes were pre-specified in the study protocol as maternal death, eclampsia, stroke, kidney injury and perinatal death. Secondary clinical outcomes were process measures (maternal magnesium sulphate administration, maternal Critical Care Unit (CCU) admission) and perinatal outcomes (stillbirth, early and late neonatal death, delivery at <34 weeks, delivery at <37 weeks). Kidney injury was defined as highest creatinine during admission ≥90 μmol/L. It was not possible to distinguish between acute kidney injury (as a consequence of pre-eclampsia) and chronic renal disease, because baseline creatinine levels were not known. Early neonatal death was defined as death in the first 7 days of life; late neonatal death was defined as death between 8 and 28 days of life. Perinatal death was defined as including stillbirth, early neonatal and late neonatal death ie, extended perinatal death [10]. Critical Care Unit admission was defined as admission to a critical care area providing at least additional monitoring and interventions [11]. Eclampsia and stroke were recorded if they occurred prior to or during admission. Maternal death was recorded if it occurred during admission only. Stillbirth may have occurred prior to admission but was only recorded when identified during admission.

For objective clinical endpoints (maternal death, eclampsia, stroke, kidney injury and perinatal death), analysis using logistic regression models for correlation between baseline and admission characteristics and these outcomes was performed, using AUROC values and 95% confidence intervals (CI). The aim of the logistic regression models was to explore which variables were most strongly associated with outcomes, rather than to create a clinical prediction model.

The study was approved by the Stellenbosch University Ethics Committee (N14/06068), University of Cape Town Ethics Committees (410/2014) and the University of the Free State Ethics Committee (230408-011). Local ethics committees at two of the three sites required individual informed written consent to be obtained before the woman was enrolled in the study (or waiver of consent was granted if the woman was unconscious). Institutional level agreement for the study was given at the third site. The study was funded by The Bill and Melinda Gates Foundation (Grant ID: OPP1086183). The funders were not involved in conducting the research or writing the paper. Patients were not involved in the designing or analysis of the study.

As an observational study, a formal power calculation was not undertaken. Intended sample sizes are outlined in the study protocol. Three large units with a high prevalence of hypertension were selected to obtain a meaningful sample to evaluate clinical associations. It was considered that the high numbers of adverse outcomes anticipated would allow accurate determination of clinically important associations. Statistical analysis was performed in the statistical package Stata (version 11.2), College Station, TX. The study is reported in accordance with STrengthening the Reporting of OBservational studies in Epidemiology (STROBE) guidelines.

## RESULTS

**Figure 1:**
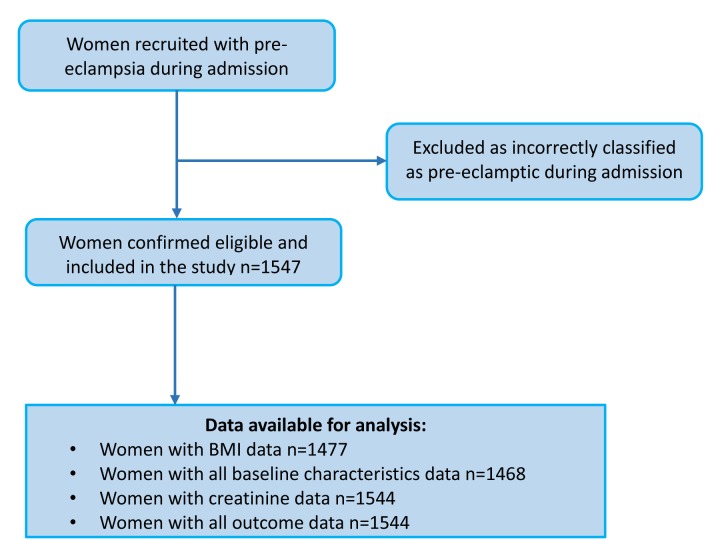
Flow diagram of participants.

A total of 1547 women with a clinical diagnosis of pre-eclampsia were eligible, consented and were included in the analysis, including 42 twin pregnancies (**Figure 1**). Comparison with expected monthly numbers of cases in these units showed that a high majority of women were recruited but formal documentation of women who declined was not made. Participant characteristics are shown in **Table 1**. The BMI in this cohort of women was high (mean BMI 30.4 kg/m^2^; SD 7.73) and the majority were multiparous (63.5%). The mean highest BP during admission was 172/104 mm Hg and 43% of the highest BPs during admission occurred in the postpartum period (**Table 1**). 89.3% of women had one plus or more dipstick proteinuria on admission; the remaining women fulfilled other pre-eclampsia diagnostic criteria or developed proteinuria whilst an inpatient. Mean gestational age at delivery was 33.4 weeks (SD 4.7); 87.8% of women had an iatrogenic onset of labour (clinician-indicated) and 69.7% of deliveries were by Caesarean section.

**Table 1:** Mean ± standard deviation or n (percentage) of demographic, admission and delivery characteristics. BMI – body mass index.

	All sites	Groote Schuur Hospital	Kimberley Hospital	Tygerberg Hospital
**Number of women**	**1547**	**770 (49.8)**	**167 (10.8)**	**610 (39.4)**
**Demographics:**				
Age at delivery, year	27.6 ± 6.2	28.0 ± 6.0	28.3 ± 7.2	27.0 ± 6.2
Body mass index, kg/m^2^	30.4 ± 7.73	31.0 ± 8.3	31.9 ± 8.3	29.2 ± 6.7
Multiparous	983 (63.5)	514 (66.8)	117 (70.1)	352 (57.7)
**Admission:**				
Gestation on admission, weeks	32.8 ± 4.9	32.0 ± 4.8	33.9 ± 4.7	33.5 ± 4.9
Systolic BP (mmHg)	150 ± 20.6	150 ± 22.3	147 ± 20.0	150 ± 18.2
Diastolic BP (mmHg)	97 ± 15.4	98 ± 15.6	92 ± 16.9	97 ± 14.3
Negative/Trace dipstick proteinuria	165 (10.7)	93 (12.1)	69 (42.6)	3 (0.5)
+1	196 (12.7)	134 (17.4)	15 (9.3)	47 (7.7)
+2	578 (37.5)	245 (31.9)	32 (19.8)	301 (49.3)
+3	601 (39.0)	296 (38.5)	46 (28.4)	259 (42.5)
24-h urine completed	379 (24.5)	170 (22.1)	23 (15.2)	186 (30.8)
24-h proteinuria (g/24 h)	1.8 ± 2.3	1.7 ± 2.3	1.4 ± 2.1	1.92 ± 2.3
**Delivery:**				
Admission to delivery, days	4.1 ± 7.3	5.1 ± 8.1	3.9 ± 9.6	3.0 ± 4.8
Gestation at delivery, weeks	33.4 ± 4.7	32.8 ± 4.5	34.5 ± 4.4	33.9 ± 4.9
Clinician-indicated (iatrogenic) delivery	1357 (87.8)	636 (82.6)	147 (88.6)	574 (94.3)
Caesarean section (pre-labour and emergency)	1060 (69.7)	549 (71.3)	115 (69.3)	417 (68.5)
**Highest BP (mmHg):**				
Systolic BP	172 ± 16.9	174 ± 17.9	171 ± 16.1	170 ± 15.7
Diastolic BP	104 ± 14.60	106 ± 15.7	102 ± 17.1	103 ± 12.2
Highest SBP taken postpartum	658 (42.9)	333 (43.3)	95 (57.6)	230 (38.2)
Time from highest (*antenatal/ intrapartum*) systolic BP to delivery, days (n = 876)	3.0 ± 5.9	3.8 ± 6.9	2.7 ± 6.36	2.1 ± 4.0
Time from delivery to highest systolic BP (*postnatal),* days (n = 658)	1.6 ± 6.1	1.2 ± 1.4	2.3 ± 2.3	2.3 ± 1.8

In this population of women with pre-eclampsia, 16 (1%) women died during their admission. Eclampsia occurred in 147 (9.5%) women and identified stroke occurred in 4 (0.3%) women. Of the 16 women who died, ten also had eclampsia. 7% of those with eclampsia died, compared to 0.4% of those without eclampsia (relative risk 15.9; 95% CI = 5.9-43.1). Three of the four women with stroke also had eclampsia and subsequently died. Most women received magnesium sulphate (86.9%). 17.6% of women had kidney injury and 3% of those with kidney injury died, compared to 0.5% of those with normal creatinine (relative risk 6.2; 95% CI = 2.2-17.8). Of 1589 births, 332 (21.0%) had a perinatal death; of these, 281 (84.5%) were stillbirths and 51 (15.4%) were neonatal deaths. Of 1308 live births, 913 (70.0%) were born preterm prior to 37 completed weeks of gestation, and 544 (41.7%) were born before 34 weeks’ gestation (**Table 2**).

**Table 2:** Mean ± standard deviation or n (percentage) of principal maternal, secondary maternal, principal perinatal, secondary perinatal and process measure outcomes

	All sites	Groote Schuur Hospital	Kimberley Hospital	Tygerberg Hospital
Number of women	**1547**	**770 (49.8)**	**167 (10.8)**	**610 (39.4)**
**Principal outcomes**				
**Maternal outcomes:**				
Maternal death	16 (1.0)	3 (0.4)	6 (3.6)	7 (1.1)
Eclampsia	147 (9.5)	71 (9.2)	16 (9.6)	60 (9.8)
Stroke	4 (0.3)	2 (0.3)	0 (0)	2 (0.3)
Highest creatinine during admission (μmol/L)	79.6 ± 78.8	86.0 ± 76.4	84.96 ± 116.40	70.1 ± 69.9
Kidney injury (n = 1544)	272 (17.6)	174 (22.6)	26 (15.9)	72 (11.8)
PERINATAL DEATH	332 (21.0)	188 (23.6)	80 (15.1)	118 (18.9)
**Secondary outcomes**				
**Process measures:**				
Maternal magnesium sulphate administration	1345 (86.9)	686 (89.1)	120 (71.9)	539 (88.4)
Maternal Critical Care Unit admission	453 (29.3)	105 (13.6)	114 (68.3)	234 (38.4)
Total number of infants	1589	793	172	624
**Perinatal outcomes:**				
Stillbirth	281 (17.7)	162 (20.4)	16 (9.3)	103 (16.5)
Early neonatal death	39 (2.5)	21 (2.6)	6 (3.5)	12 (1.9)
Late neonatal death	12 (0.8)	5 (0.6)	4 (2.3)	3 (0.5)
Preterm birth <37 weeks	913 (70.0)	491 (78.1)	99 (63.5)	323 (62.1)
Preterm birth <34 weeks	544 (41.7)	303 (48.2)	52 (33.3)	189 (36.3)

Using stepwise logistic regression modelling, the association between baseline and admission characteristics and the principal clinical outcomes was evaluated, including eclampsia, kidney injury and perinatal death (**Table 3**). Due to small numbers, maternal death and stroke were excluded from this analysis.

**Table 3:** Stepwise logistic regression model AUROC values and 95% confidence intervals for the association between baseline and admission characteristics and the three principle outcomes (eclampsia, kidney injury and perinatal death)*

Predictors	Eclampsia		Kidney injury		Perinatal death	
**Total number of women**	**1460**		**1459**		**1500**	
	**AUROC**	**95% CI**	**AUROC**	**95% CI**	**AUROC**	**95% CI**
Prediction model	0.79	0.75, 0.83	0.68	0.64, 0.72	0.81	0.79, 0.84
Advanced maternal age	**0.76** ↓	**0.71, 0.80**	0.52	0.48, 0.56	0.46	0.43, 0.50
High Body mass index	0.64 ↓	0.59, 0.69	0.46	0.42, 0.50	0.50	0.46, 0.54
Multiparity	0.67 ↓	0.63, 0.71	0.53	0.50, 0.56	0.56	0.53, 0.58
Low gestation at admission	0.59	0.54, 0.64	0.58	0.54, 0.62	**0.81**	**0.77, 0.84**
High admission systolic BP	0.52	0.47, 0.58	0.55	0.51, 0.59	0.52	0.49, 0.56
High admission diastolic BP	0.53	0.47, 0.58	0.57	0.53, 0.62	0.51	0.47, 0.55
High highest systolic BP	0.54	0.49, 0.59	**0.64**	**0.60, 0.68**	0.46	0.42, 0.50
High diastolic BP at highest systolic BP	0.51	0.46, 0.57	0.59	0.55, 0.63	0.48	0.44, 0.52
High admission dipstick proteinuria	0.59	0.54, 0.64	0.59	0.56, 0.63	0.54	0.51, 0.58

CI – confidence interval, AUROC – area under the receiver operating characteristic curve

*Most important characteristic for each outcome shown in bold. Direction of association as stated in first column, unless an arrow present (↓), indicating association in opposite direction

Young maternal age was the strongest single predictor of eclampsia (AUROC = 0.76, 95% CI = 0.71-0.80); women younger than 20 years had a 30% risk of eclampsia whilst women of 30 years or older had a 3.5% risk of eclampsia. The association between young maternal age and eclampsia occurred despite mean admission SBP and highest SBP during admission significantly increasing with advancing maternal age. The unadjusted odds ratio for eclampsia in women younger than 20 years, compared to those aged 20-29 years, was 4.52 (95% CI = 3.02-6.76). Following adjustment for admission and highest BP, the odds ratio was 5.04 (95% CI = 3.34-7.61). Therefore, BP works against the association between age and eclampsia, but only weakly. There was a 40% risk of eclampsia in women with BMI less than 18.5 kg/m^2^; in women with BMI≥35 kg/m^2^, the risk of eclampsia was 5.6% (AUROC = 0.64, 95% CI = 0.59-0.69). BP variables were not significant predictors on their own for risk of eclampsia.

For eclampsia, the model including maternal age, BMI, gestational age at admission, highest SBP, and admission dipstick proteinuria, gave an AUROC value of 0.79 (95% CI = 0.75-0.83). Maternal age and nulliparity conveyed similar risk of eclampsia as measured by changes in the log likelihood, with young women and nulliparous women being at higher risk; therefore, only young maternal age (the stronger of the two predictors) was necessary in the model. Although BP variables were not significantly associated with eclampsia individually, highest SBP was included in the model as it was significant after adjustment.

For kidney injury, highest SBP had the strongest association (AUROC = 0.64, 95% CI = 0.60-0.68). For women with a highest SBP≥200 mm Hg, 40% had kidney injury. When a change in SBP (from admission to highest SBP) was compared to highest SBP alone, highest SBP performed better than change in SBP. For prediction of kidney injury, a model including gestational age at admission, admission SBP and DBP, highest SBP, and admission dipstick proteinuria gave an AUROC value of 0.68 (CI = 0.64-0.72).

Gestation at admission was the most strongly associated variable with perinatal death (AUROC = 0.81, 95% CI = 0.77-0.84), with early gestation strongly associated with perinatal death. Between 26 and 27 completed weeks of pregnancy, risk of perinatal death was 50%; from 32 weeks’ gestation, risk of perinatal death was approximately ≤10%. All BP variables were statistically not significantly associated with perinatal death.

For perinatal death, the model, including gestational age at admission, admission SBP and admission dipstick proteinuria, gave an AUROC value of 0.81 (0.79-0.84). Again, although BP variables were not statistically significantly associated with perinatal death as individual variables, highest SBP was included in the model as it was significant after adjustment.

## DISCUSSION

### Main findings

In this South African cohort with pre-eclampsia managed in tertiary facilities, severe hypertension and pre-eclampsia complications were common. Young maternal age was strongly associated with risk of eclampsia, highest SBP was associated with kidney injury and early gestation at admission was associated with perinatal death.

### Strengths and limitations

This large, multi-centre, prospective study of women with pre-eclampsia managed in South African tertiary facilities explored the incidence of complications and their association with baseline and admission characteristics. Although women in secondary level facilities may not have severe disease, this cohort is representative of referral facilities that manage women with mild to severe pre-eclampsia.

The vital signs included in analysis were taken at and following admission to tertiary care, with many having already received treatment. It was not feasible to collect accurate temporal data on antihypertensive and magnesium sulphate use and adverse outcomes due to minimal documentation. Therefore, assessing for effects of intervention and determining the temporal association between interventions and outcomes, including eclampsia and stillbirth, was not possible.

Severe hypertension and pre-eclampsia complications were common, despite access to antihypertensive therapy, magnesium sulphate administration and critical care. Despite referral to tertiary-level care, the eclampsia incidence in our cohort (9.5%) was much higher than the incidence of eclampsia in the MAGPIE trial low-income country pre-eclampsia cohort (2.3%) [12]. The MAGPIE trial eclampsia incidence may have been underestimated as only hospital data were used, excluding women giving birth outside facilities. In our setting, most births occur at facility-level and our incidence may be more reflective of the true incidence of eclampsia in South Africa. However, in our study it was not possible to distinguish between eclampsia that occurred prior to tertiary care and eclampsia that occurred whilst receiving tertiary care, due to limitations with patient notes documentation. The eclampsia incidence may appear higher than expected for women receiving adequate care because the eclampsia may have occurred prior to that care.

Stroke occurred in 0.3% of women in this cohort, in association with a high incidence of severe systolic hypertension. The association between systolic hypertension and pre-eclampsia-related stroke was shown in a small cohort study of 28 women with pre-eclampsia-related stroke, with 95.8% of women with BP measurements taken immediately prior to the stroke having a systolic BP≥160mmHg [13]. This study has been criticised due to small sample size, lack of statistical analysis confirming the independent association of SBP and potentially biased case ascertainment [14]. However, the low incidence of stroke in our cohort may reflect an underestimation of stroke incidence, with stroke going unrecognised unless severe, categorised as eclampsia or missed when occurring postnatally away from facility. Conversely, it may be the true incidence and demonstrate good management of severe systolic hypertension once in tertiary care.

Stillbirth was more common in our cohort (17.7%) than the recent WHO multi-country survey’s stillbirth rate of 6.36% in women with pre-eclampsia [15]. The high stillbirth rate in our cohort may reflect inadequate primary and secondary antenatal care. Most livebirths within our cohort were preterm, iatrogenic and delivered by Caesarean section. This may reflect appropriate obstetric care in response to pre-eclampsia complications and good neonatal facilities once receiving tertiary care. There were relatively few neonatal deaths, suggesting prematurity was not a major contributor to perinatal mortality in this setting.

Current literature suggests advanced maternal age, obesity and black ethnicity are associated with risk of pre-eclampsia [16-22]. In our cohort, despite BP increasing with advancing maternal age, younger women were at higher risk of eclampsia. Similar associations were shown in the WHO multi-country survey; adolescents of <17 years were more likely to develop eclampsia (adjusted odds ratio 1.73; 95% CI = 1.23-2.43), despite no association between young age and pre-eclampsia [15]. Retrospective studies of women with eclampsia in Nigeria and USA also support the association between eclampsia and young maternal age [23,24]. The fullPIERS and miniPIERS models showed no association between maternal age and eclampsia, although eclampsia was rare [25,26]. These and our findings suggest that although advanced maternal age is a risk factor for pre-eclampsia, teenagers are at higher risk of eclampsia, even when referred to tertiary care.

The association between high BMI and risk of pre-eclampsia is widely acknowledged [16-22]. The cohort design of our study does not allow robust conclusions on this association, although the data suggests raised BMI is a risk factor for pre-eclampsia. Our study demonstrates an association between low BMI and eclampsia. This is a novel finding and may be related to the association between eclampsia and young age. Existing literature, although limited, suggests that weight gain during pregnancy is more critical for teenage mothers than for older mothers [27,28]. In those teenage mothers with low BMI, it is plausible that the woman competes with the growing fetus for nutrients and, for this reason, these women may be more at risk of adverse outcomes.

Almost one fifth of women in our cohort had evidence of kidney injury. Systolic hypertension was strongly associated with risk of kidney injury and kidney injury was associated with a six times higher risk of maternal death. Proteinuria was less strongly associated with risk of kidney injury. 76.5% of women had 2+ or more proteinuria. Although not as robust as quantitative assessment, this degree of proteinuria is likely to be an accurate reflection of kidney pathology. It was not possible to distinguish between acute and chronic renal impairment because few women had baseline creatinine values; however, previous studies have shown that the majority were likely to reflect acute kidney injury secondary to pre-eclampsia [29-31]. Little exists in the literature on the long-term morbidity associated with pre-eclampsia-related acute kidney injury, particularly in LMICs where the burden is greatest. The importance of BP control in preventing acute kidney injury in a pre-eclamptic population requires further evaluation. Our data are consistent with the observation that chronic kidney disease affects almost twice as many women of childbearing age in low-income countries compared to high-income countries [29]. Obstetric acute kidney injury may be contributing to this discrepancy.

Our study showed a strong association between early gestational age at admission and risk of perinatal death. This association has been demonstrated with regard to stillbirth specifically, in a Haitain cohort of women with pre-eclampsia [32]. Early-onset disease is known to be severe and women may present too late for management to influence perinatal outcomes in our settings. The focus should be on community detection of pre-eclampsia and timely referral. Once in tertiary care, prolonging pregnancy may be a key intervention to improve perinatal outcomes but must be balanced with maternal morbidity. Given the high mortality antepartum and the low neonatal death rates, in lower-resourced settings, where monitoring during expectant management may be more challenging, risks of prematurity related to induction of labour to avoid stillbirth require further prospective evaluation.

### Interpretation

Severe hypertension was common in this cohort, despite adequate access to appropriate antihypertensive treatment. This may, in part, be explained by the use of BP devices validated in pregnancy, including pre-eclampsia (rare in previous literature; most devices under-read in pre-eclampsia). Consequently, this study may have correctly identified hypertension that would have previously gone unrecognised [33]. The high incidence of severe hypertension may also represent under-treatment of severe hypertension by clinicians. As women are often asymptomatic and appear well, it is easy to underestimate their risk of complications. This study highlights the need to proactively control severe hypertension.

The incidence of pre-eclampsia complications, perinatal death and preterm delivery was much higher than reported in other low- and middle-income studies and despite access to tertiary care interventions. This may solely be a reflection of a cohort of women unwell enough to warrant tertiary care (and therefore, be at greatest risk of complications) or it could be explained by poor access to antenatal and postnatal care outside of facilities, socioeconomic factors resulting in delays in seeking care or pathophysiological factors inherent to the population.

Whether the association between teenage pregnancy and risk of eclampsia can be attributed to concomitant socioeconomic factors or biological features of young maternal age is contentious [34]. It has been explained by socioeconomic factors, with younger women attending antenatal care less frequently and seeking care less readily when symptomatic. Conversely, although the pathophysiological mechanisms are poorly understood [24], the association has been suggested to be explained by a biological immaturity, also related to brief paternal exposure, and lability of the central nervous system in younger women [15]. Teenage mothers are also more likely than older mothers to have inadequate weight gain during pregnancy and be nulliparous, which may result in inadequate immunological adaptation. These factors may accelerate the biological association between young age and eclampsia. Studies from USA, Canada and the Netherlands, as well as the most recent UK and Ireland Confidential Enquiries into Maternal Deaths (MBRACE) report, have shown that those of African ethnicity have increased risk of pre-eclampsia complications, including maternal death, despite living in high-income countries [21,35-42].

## CONCLUSIONS

The results from this study could be used to inform management guidelines and policy. Delays in identifying the severity of hypertension will impact on timely referral and intervention and the outcomes. The association between teenage pregnancy and eclampsia risk highlights the importance of antenatal care, prompt referral and pregnancy education, including danger signs of imminent eclampsia, for young mothers.
